# Elevated ApoB/ApoA ratio is associated with increased risk of sudden sensorineural hearing loss

**DOI:** 10.3389/fendo.2026.1955992

**Published:** 2026-10-15

**Authors:** Zhisong Yan, Weilin Chen, Xiaoyang Cao, Xiaodong Chen

**Affiliations:** Department of Otolaryngology, Tongzhou Branch of Nantong People’s Hospital (Nantong Tongzhou People’s Hospital), Nantong, Jiangsu, China

**Keywords:** ApoB/ApoA ratio, biomarker, inverse probability of treatment weighting, lipid metabolism, propensity score matching, sudden sensorineural hearing loss

## Abstract

**Objective:**

To investigate the association between the apolipoprotein B to apolipoprotein A (ApoB/ApoA) ratio and sudden sensorineural hearing loss (SSNHL) risk in Nantong, China.

**Methods:**

This cross-sectional study included 1211 participants (674 with normal hearing, 537 with SSNHL). Baseline demographics, comorbidities, and biochemical indicators were compared. Multivariable logistic regression evaluated the independent association between the ApoB/ApoA ratio and SSNHL risk. Inverse probability of treatment weighting (IPTW) and propensity score matching (PSM) were applied for sensitivity analysis. Stratified and dose-response analyses were also conducted.

**Results:**

The groups differed significantly in age, sex, hypertension, BMI, and various metabolic markers (all P < 0.05). In the fully adjusted model, an elevated ApoB/ApoA ratio was significantly associated with increased SSNHL risk (OR = 9.34, 95% CI: 3.93-22.21). This positive association remained robust after PSM (OR = 8.79, 95% CI: 3.26-23.66). IPTW analysis showed the high-ratio group (0.78-1.34) had a higher SSNHL risk than the low-ratio group (0.13-0.58) (OR = 2.28, 95% CI: 1.79-2.90). Dose-response analysis indicated a significant linear positive correlation between the ratio and SSNHL prevalence, both before and after PSM. Stratified analyses demonstrated this association remained consistent across age, sex, hypertension, and diabetes subgroups (all P for interaction > 0.05).

**Conclusion:**

A high ApoB/ApoA ratio has a significant linear positive association with increased SSNHL risk. It serves as an independent clinical biomarker for SSNHL risk assessment, providing a basis for metabolic management.

## Introduction

Hearing loss has become a very common sensory disorder globally and is the fourth leading cause of disability (1). It is reported that approximately 20% of the global population suffers from hearing loss (2). By 2030, hearing loss will become one of the main causes of the global burden of disease (2). The social and economic burden caused by hearing loss is enormous. Previous studies have shown that when calculating the monetary value of years lived with disability due to hearing loss, the annual global cost exceeds 750 billion USD (3).

Sudden sensorineural hearing loss (SSNHL) is a common and destructive clinical emergency among hearing loss conditions. Its exact pathogenesis has not yet been fully elucidated. Current mainstream pathological hypotheses include viral infection, microcirculatory dysfunction, autoimmune reactions, and metabolic abnormalities (4). Notably, the blood supply to the inner ear mainly relies on the labyrinthine artery, a terminal vessel. Due to the lack of collateral circulation, it is extremely sensitive to ischemia, hypoxia, and microvascular lesions (5, 6). However, as pointed out by top guidelines in the cardiovascular field, routine clinical lipid testing indicators (such as relying solely on total cholesterol or triglycerides) often lack sufficient sensitivity and comprehensiveness when evaluating vascular ischemic events and residual risks (7). This limitation also exists when evaluating inner ear microvascular ischemia (8, 9).

Apolipoprotein B (ApoB) is the main structural protein of almost all atherogenic lipoproteins (such as LDL, VLDL) and reflects the total number of harmful lipoprotein particles that promote lipid deposition in the vascular wall. Meanwhile, apolipoprotein A (ApoA, mainly ApoA) is the core component of high-density lipoprotein (HDL), responsible for anti-atherosclerotic reverse cholesterol transport. Therefore, the ApoB/ApoA ratio can more accurately reflect the dynamic balance between “atherogenic” and “anti-atherosclerotic” lipoproteins in the body (10). Multiple clinical studies have confirmed that the ApoB/ApoA ratio is significantly better than traditional single lipid indicators in predicting cardiovascular and cerebrovascular events, endothelial dysfunction, and peripheral microvascular complications (11, 12). Despite this, the potential pathological link between this ratio and the risk of developing SSNHL has not been fully explored or elucidated.

Based on these research gaps, this study aims to explore the independent association between the ApoB/ApoA ratio and the risk of SSNHL in the population of Nantong, China. By using large-scale cross-sectional data and comprehensively applying statistical methods such as propensity score matching (PSM) and inverse probability of treatment weighting (IPTW) to strictly control potential confounders, this study expects to verify whether the ApoB/ApoA ratio can be used as a reliable clinical biomarker to assess the clinical association with SSNHL. This will provide new epidemiological evidence for metabolic risk stratification and management of hearing loss.

## Materials and methods

### Study population and design

This is a retrospective cross-sectional study. The reporting of this study adheres to the Strengthening the Reporting of Observational Studies in Epidemiology (STROBE) reporting guideline for cross-sectional studies. Participants were selected from individuals who visited the Nantong Tongzhou People’s Hospital, Jiangsu Province, China, between July 2017 and December 2025.

### Inclusion criteria

1). Age between 20 and 85 years. 2). Diagnosed with unilateral or bilateral sudden sensorineural hearing loss (SSNHL) by a certified otolaryngologist through clinical examination and standardized pure-tone audiometry (PTA) evaluation. The diagnosis strictly adhered to established clinical practice guidelines, defined as a sensorineural hearing loss of 30 dB or greater over at least three contiguous audiometric frequencies, occurring within a 72-hour period (hearing loss group). Furthermore, regarding spontaneous recovery, patients who had experienced complete spontaneous recovery prior to their initial audiological evaluation were not included in this study cohort, whereas those with partial or no recovery upon admission were included and evaluated. 3). Individuals who underwent routine annual health examinations for occupational or general health screening at the same medical institution during the same period, representing the same catchment population (normal hearing group). Due to the nature of the electronic health screening system, their hearing status was recorded and extracted as a binary diagnostic classification (normal vs. abnormal). All control subjects were clinically certified as having a “normal” hearing status and were confirmed to be free of previous hearing loss and any active otological diseases. 4). Complete baseline demographic information, medical history, and comprehensive laboratory biochemical data (especially ApoB and ApoA).

### Exclusion criteria

1). Conductive or mixed hearing loss. 2).Hearing damage caused by otitis media, Meniere’s disease, acoustic neuroma, ear trauma, or severe noise exposure. 3).Patients with severe cardiac dysfunction, active malignancies, severe hepatic or renal failure, or autoimmune diseases. 4). Patients who recently took large amounts of medications affecting lipid metabolism.

After strict screening based on the above criteria, a total of 1211 subjects were finally included, consisting of 674 individuals with normal hearing and 537 SSNHL patients. The study protocol was reviewed and approved by the Ethics Committee of Nantong Tongzhou People’s Hospital (Ethics Approval Number: 2026-Y028). All participants or their legal representatives signed informed consent forms.

### Data collection and laboratory

Measurements Baseline demographic information and clinical characteristics of all participants were collected through the electronic medical record (EMR) system, including age, sex, body mass index (BMI), and medical history such as hypertension or diabetes.

Venous blood samples were collected in the early morning after an overnight fast of at least 8 hours for laboratory biochemical analysis. Tested indicators included blood urea nitrogen (BUN), uric acid (UA), total cholesterol, total bilirubin (TBIL), triglycerides (TG), creatinine (Cre), fasting plasma glucose (FPG), alanine aminotransferase (ALT), aspartate aminotransferase (AST), apolipoprotein A (ApoA), and apolipoprotein B (ApoB). All biochemical indicators, including ApoB and ApoA, were measured using standard automated biochemical analyzers via the immunoturbidimetric assay method in our hospital’s central laboratory.

The ApoB/ApoA ratio was calculated for each participant and evaluated as a continuous variable in the statistical analysis. It was also converted into a categorical variable based on tertiles: the low-ratio group (0.13 - 0.58), the medium-ratio group (0.58 - 0.78), and the high-ratio group (0.78 - 1.34), for subsequent calculation and comparison.

### Statistical analysis

A P-value < 0.05 was considered statistically significant. All analyses were performed using Empower software and R version 4.2.0.

Baseline characteristics: Continuous variables were expressed as mean ± standard deviation (Mean ± SD), and comparisons between groups (normal hearing group vs. hearing loss group) were performed using independent sample t-tests. Categorical variables were expressed as frequencies (percentages, %), and group comparisons were performed using Chi-square tests.

Multivariable regression analysis: A multivariable logistic regression model was used to evaluate the independent association between the ApoB/ApoA ratio and hearing loss risk, calculating odds ratios (OR) and their 95% confidence intervals (CI). Three stepwise adjusted models were constructed. Model 1 was an unadjusted crude model. Model 2 was adjusted only for age and sex. Model 3 was fully adjusted for all baseline covariates in **Table 1** (including BUN, UA, Cholesterol, TBIL, TG, Cre, FPG, ALT, AST, BMI, hypertension, and diabetes).

**Table 1:** Baseline data for populations in local regions of China.

HL	Normal	Hearing loss	P-value
N	674	537	
Age(years)	53.52 ± 13.32	56.30 ± 13.82	<0.001
BUN(mmol/L)	6.49 ± 3.48	6.11 ± 1.73	0.023
UA(umol/L)	349.20 ± 97.45	308.41 ± 95.90	<0.001
Cholesterol(mmol/L)	5.06 ± 1.00	5.07 ± 0.99	0.903
TBIL(μmol/L)	12.07 ± 5.40	14.17 ± 5.95	<0.001
TG(mmol/L)	1.59 ± 0.78	1.27 ± 0.88	<0.001
Cre(μmol/L)	97.43 ± 71.55	57.34 ± 15.04	<0.001
FPG(mmol/L)	6.04 ± 1.77	6.19 ± 2.03	0.158
ALT (U/L)	22.80 ± 15.57	22.90 ± 21.47	0.93
AST (U/L)	20.92 ± 9.06	23.00 ± 12.32	<0.001
BMI(kg/m2)	24.77 ± 3.69	24.01 ± 3.35	<0.001
ApoA	1.38 ± 0.31	1.53 ± 0.44	<0.001
ApoB	0.92 ± 0.20	0.97 ± 0.28	0.004
ApoB/ApoA	0.69 ± 0.21	0.68 ± 0.25	0.038
Gender(%)			<0.001
Male	442 (65.58%)	258 (48.04%)	
Female	232 (34.42%)	279 (51.96%)	
High Blood Pressure(%)			<0.001
No	386 (57.27%)	432 (80.45%)	
Yes	288 (42.73%)	105 (19.55%)	
Diabetes(%)			0.456
No	524 (77.74%)	427 (79.52%)	
Yes	150 (22.26%)	110 (20.48%)	

HL, Hearing loss, N, Number, BUN, Blood Urea Nitrogen, UA, Uric Acid, TBIL, Total Bilirubin, TG, Triglycerides, Cre, Creatinine, FPG, Fasting Plasma Glucose, ALT, Alanine Aminotransferase, AST, Aspartate Aminotransferase, BMI, Body Mass Index, APOB, Apolipoprotein B, APOA, Apolipoprotein A.

Propensity Score Matching (PSM) and Inverse Probability of Treatment Weighting (IPTW): To maximize the control of potential confounding bias in observational studies and simulate the effects of a randomized controlled trial, this study further used PSM and IPTW for sensitivity analysis. The propensity score for each patient was calculated based on all baseline covariates. IPTW (Model 4) was used to weight different ApoB/ApoA tertile groups to balance baseline differences. PSM was performed using nearest-neighbor matching at a 1:1 ratio (caliper value set at 0.05). Recognizing that strict balance for all continuous metabolic variables might not be fully achieved by matching alone, we adopted a doubly robust estimation strategy. In Model 5, the risk was re-evaluated within this matched cohort using a secondary multivariable logistic regression that further adjusted for all baseline covariates (the same variables as Model 3) to eliminate any persistent residual confounding.

Subgroup and interaction analysis: Stratified analyses were performed by age (20–54 years, 55–85 years), sex (male, female), hypertension (yes, no), and diabetes (yes, no). A likelihood ratio test was used to evaluate whether there was a statistical interaction between the stratifying variables and the ApoB/ApoA ratio.

Dose-Response Relationship: Smooth curve fitting (based on a generalized additive model) was used in both the original full cohort and the PSM-matched cohort to flexibly assess the linear or non-linear association between the continuous ApoB/ApoA ratio and the prevalence of hearing loss. All statistical tests were two-sided.

## Results

### Baseline characteristics

This study finally included 1211 eligible subjects, comprising 674 cases in the normal hearing control group and 537 patients with SSNHL. Baseline clinical characteristics showed statistically significant differences between the hearing loss group and the normal hearing group in terms of age, sex distribution, hypertension prevalence, and body mass index (BMI) (all P < 0.05). For laboratory biochemical indicators, the levels of uric acid (UA), total bilirubin (TBIL), triglycerides (TG), creatinine (Cre), and aspartate aminotransferase (AST) were significantly higher in the hearing loss group than in the normal control group (all P < 0.05). Regarding the apolipoprotein profile, the hearing loss group had higher unadjusted crude baseline levels of both ApoB (0.97 ± 0.28 vs 0.92 ± 0.20, P = 0.004) and ApoA (1.53 ± 0.44 vs 1.38 ± 0.31, P<0.001). Consequently, the crude unadjusted ApoB/ApoA ratio was slightly lower in the hearing loss group (0.68 ± 0.25 vs 0.69 ± 0.21, P = 0.038) (**Table 1**). However, as demonstrated in the subsequent multivariable analyses, this crude inverse distribution was severely distorted by negative confounding from other imbalanced baseline covariates.

### Multivariable regression and sensitivity analysis

To assess the association between the ApoB/ApoA ratio and hearing loss risk, multivariable logistic regression models were applied (**Table 2**). In Model 1 (unadjusted) and Model 2 (adjusted only for age and sex), an elevated ApoB/ApoA ratio was associated with an increased risk of SSNHL. In Model 3, which fully adjusted for all baseline covariates from **Table 1**, treating the ApoB/ApoA ratio as a continuous variable showed a significant positive association with hearing loss risk (OR = 9.34, 95% CI: 3.93 - 22.21). The striking shift from a slightly negative crude association (**Table 1** and Model 1) to a highly significant positive adjusted effect (Model 3) indicates a strong statistical suppression effect (negative confounding). Potent baseline confounders severely masked the true association in the univariate analysis; once strictly adjusted, the independent pathogenic contribution of the ApoB/ApoA ratio was profoundly unmasked. It is important to note that this OR represents the risk associated with a full one-unit increase in the ApoB/ApoA ratio. Given that the observed range of the ratio in our study population is approximately 0.13 to 1.34, a one-unit increase represents a substantial physiological shift across nearly the entire distribution. For sensitivity analyses, IPTW analysis treating the ApoB/ApoA ratio as a categorical variable (Model 4) showed that compared to the low-ratio group (0.13 - 0.58), the high-ratio group (0.78 - 1.34) had an increased risk of hearing loss (OR = 2.28, 95% CI: 1.79 - 2.90). In the 1:1 PSM cohort (Model 5), a doubly robust adjustment was applied to overcome the residual covariate imbalances observed post-matching (**Supplementary Table 1**). After secondarily adjusting for all covariates within the matched cohort, the continuous ApoB/ApoA ratio maintained a strong and significant positive association with SSNHL risk (OR = 8.79, 95% CI: 3.26 - 23.66).

**Table 2:** Association between ApoB/ApoA and SSNHL risk: multivariable logistic regression analysis.

Characteristic	Model 1 OR (95% CI)	Model 2 OR (95% CI)	Model 3 OR (95% CI)	Model 4 OR (95% CI)	Model 5 OR (95% CI)
ApoB/ApoA	0.77 (0.46, 1.27)	0.95 (0.57, 1.60)	9.34 (3.93, 22.21)	–	8.79 (3.26, 23.66)
Categories					
Low(0.13-0.58)	1	1	1	1	–
Middle(0.58-0.78)	0.58 (0.44, 0.77)	0.63 (0.47, 0.84)	1.12 (0.75, 1.69)	0.95 (0.76, 1.20)	–
High(0.78-1.34)	0.64 (0.48, 0.84)	0.73 (0.55, 0.97)	2.31 (1.47, 3.64)	2.28 (1.79, 2.90)	–

Model 1= No covariates were adjusted.

Model 2= Model 1+ age, gender.

Model 3 was adjusted for all covariates in **Table 1**.

Model 4 was adjusted for the same variables as Model 3 with IPTW.

Model 5 was adjusted for the same variables as Model 3 with PSM.

### Dose-response relationship

The dose-response analysis demonstrated a significant linear upward relationship between the ApoB/ApoA ratio and the risk of developing SSNHL, both in the original full cohort (**Figure 1**) and the PSM-matched cohort (**Figure 2**).

**Figure 1:**
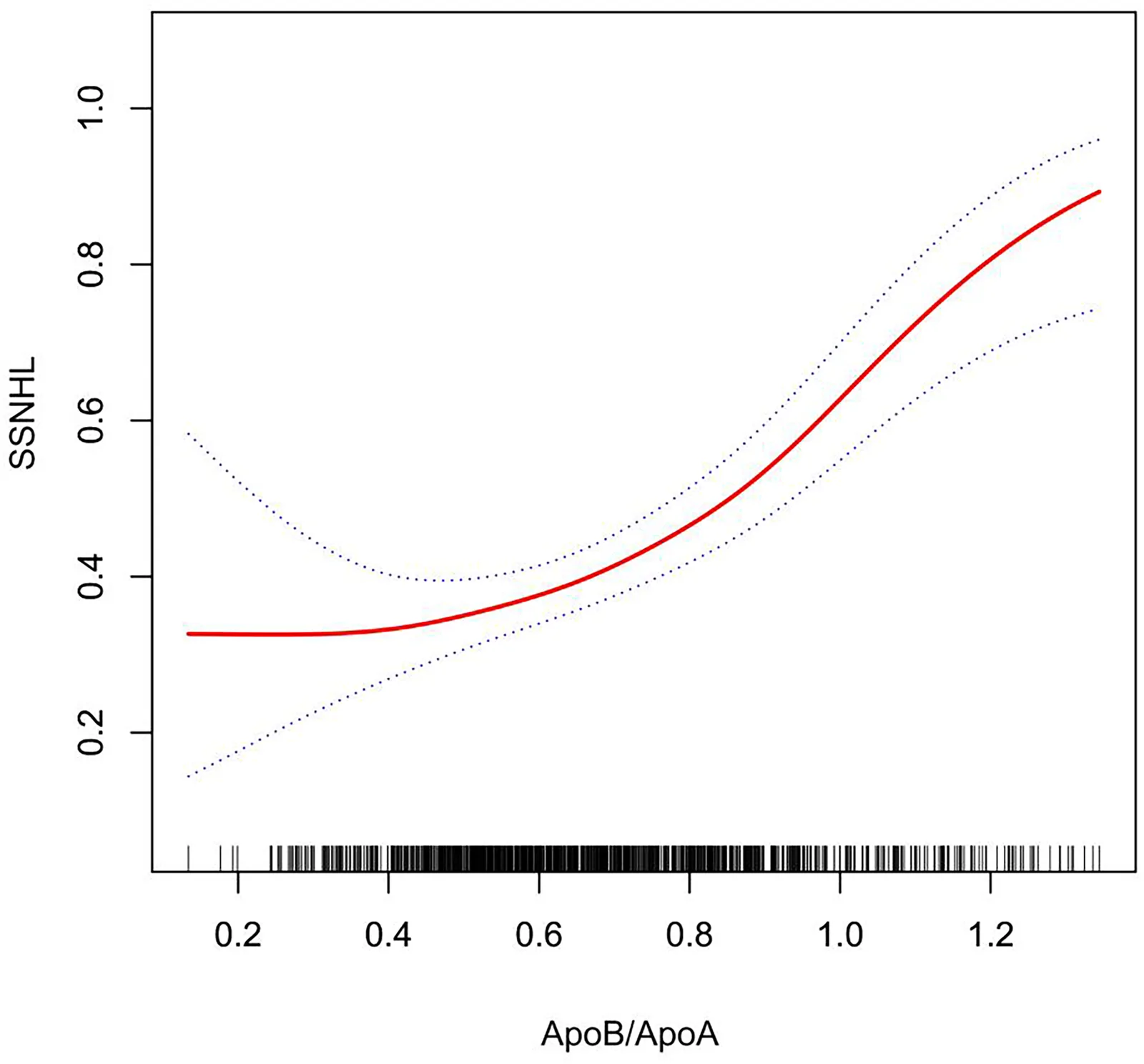
Dose-response relationship between the ApoB/ApoA ratio and the probability of SSNHL. The relationship was assessed using smooth curve fitting based on a generalized additive model. The solid red line represents the estimated probability of developing sudden sensorineural hearing loss (SSNHL), and the dotted blue bands represent the 95% confidence intervals (CI). The rug plot on the X-axis illustrates the density distribution of the participants’ ApoB/ApoA ratios.

**Figure 2:**
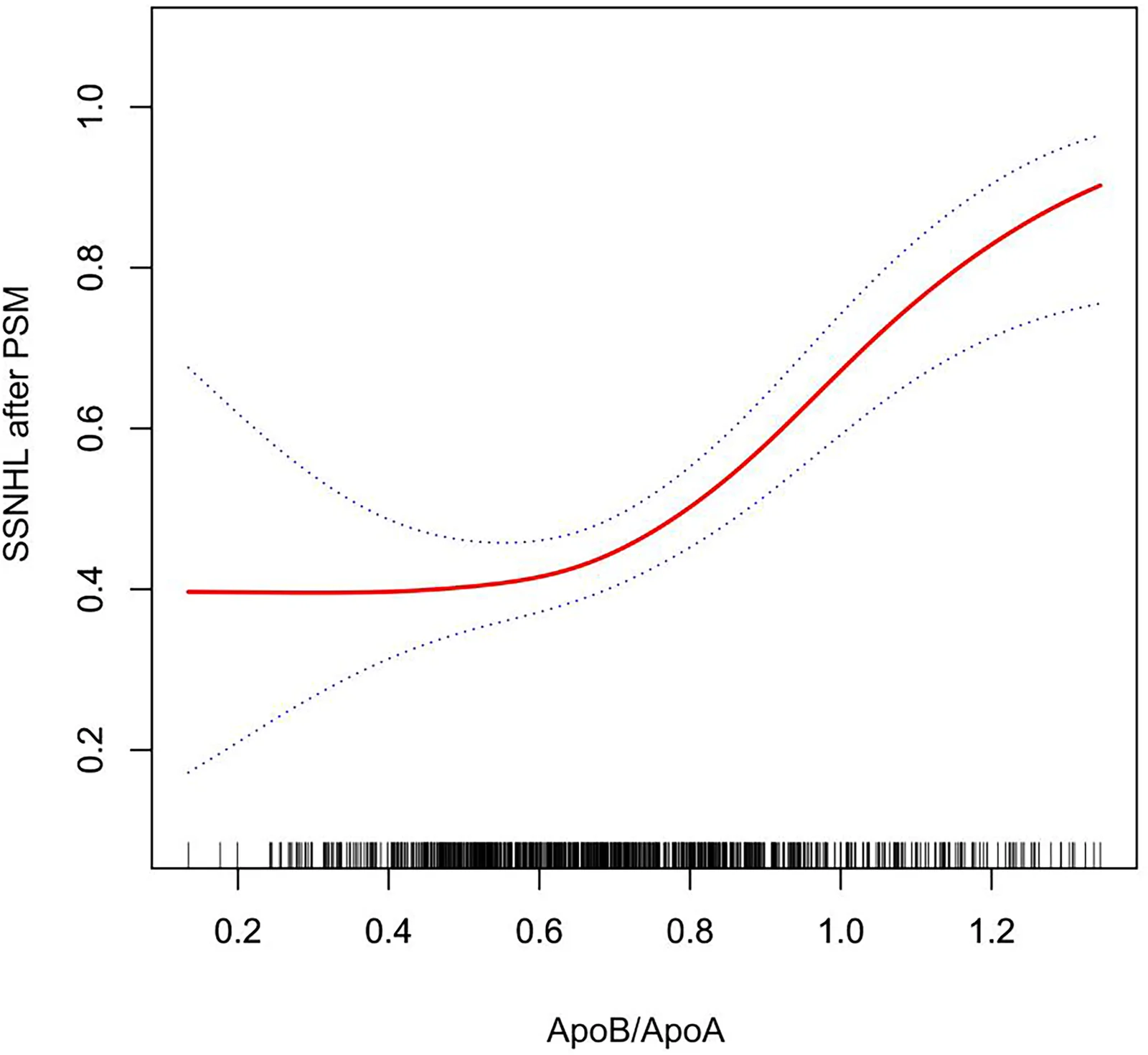
Dose-response relationship between the ApoB/ApoA ratio and the probability of SSNHL after Propensity Score Matching (PSM). The relationship was evaluated in the matched cohort using smooth curve fitting based on a generalized additive model. The solid red line indicates the estimated probability, and the dotted blue bands represent the 95% confidence intervals (CI). The rug plot on the X-axis illustrates the density distribution of the ApoB/ApoA ratios.

### Subgroup analysis

To explore whether the association between the ApoB/ApoA ratio and SSNHL risk differs among populations with different clinical characteristics, stratified analyses were performed (**Table 3**).

**Table 3:** Stratified analysis of the association between ApoB/ApoA and SSNHLrisk.

Characteristic	N	N	Model 1 OR (95% CI)	Model 2 OR (95% CI)	Model 3 OR (95% CI)	P for interaction
Stratified by age(years)						0.69
20-54	318	204	0.69 (0.31, 1.56)	1.17 (0.50, 2.76)	10.26 (2.61, 40.25)	
55-85	356	333	0.85 (0.45, 1.64)	0.85 (0.44, 1.65)	20.56 (5.72, 73.84)	
Stratified by gender						0.17
Male	442	258	0.65 (0.32, 1.32)	0.79 (0.38, 1.64)	16.81 (4.30, 65.80)	
Female	279	232	1.34 (0.63, 2.85)	1.34 (0.63, 2.86)	6.58 (1.97, 21.97)	
Stratified by hypertension						0.07
No	432	386	0.63 (0.34, 1.13)	0.75 (0.41, 1.39)	12.16 (4.16, 35.58)	
Yes	288	105	1.97 (0.69, 5.64)	3.28 (1.08, 9.94)	7.96 (1.45, 43.74)	
Stratified by diabetes						0.58
No	427	524	0.71 (0.40, 1.29)	0.87 (0.47, 1.58)	8.45 (3.30, 21.64)	
Yes	150	110	1.00 (0.36, 2.72)	1.51 (0.52, 4.41)	23.53 (1.55, 357.16)	

Age stratification: An elevated ApoB/ApoA ratio was significantly associated with an increased risk of SSNHL in both the young/middle-aged group (20–54 years) (OR = 10.26, 95% CI: 2.61-40.25) and the middle-aged/elderly group (55–85 years) (OR = 20.56, 95% CI: 5.72-73.84).

Sex stratification: The significant positive association between the ApoB/ApoA ratio and hearing loss risk was maintained in both male (OR = 16.81, 95% CI: 4.30-65.80) and female (OR = 6.58, 95% CI: 1.97-21.97) subjects.

Underlying disease stratification: Regardless of whether patients had hypertension (with hypertension: OR = 7.96, 95% CI: 1.45-43.74; without hypertension: OR = 12.16, 95% CI: 4.16-35.58) or diabetes (with diabetes: OR = 23.53, 95% CI: 1.55-357.16; without diabetes: OR = 8.45, 95% CI: 3.30-21.64), the association between a high ApoB/ApoA ratio and an increased risk of SSNHL remained consistent.

Furthermore, interaction analysis revealed no significant statistical interactions between the ApoB/ApoA ratio and stratifying variables, including age (P for interaction = 0.69), sex (P for interaction = 0.17), hypertension (P for interaction = 0.07), and diabetes (P for interaction = 0.58). This indicates that the positive correlation between the ApoB/ApoA ratio and SSNHL risk is stable and does not change significantly based on age, sex, or routine baseline metabolic disease status.

## Discussion

Based on a relatively large sample of clinical data and combined with IPTW and PSM models, this study systematically evaluated the association between lipid metabolism markers and SSNHL. It yielded two core findings: First, an elevated ApoB/ApoA ratio is an independent risk factor for SSNHL, and there is a significant continuous linear dose-response relationship between the two. Second, rigorous stratified analyses and interaction tests confirmed that this positive association remains highly stable and consistent across different age groups, sexes, and regardless of whether patients have hypertension or diabetes. These two major findings not only reveal the key role of lipid homeostasis imbalance in the pathological process of SSNHL, but also prove that the ApoB/ApoA ratio can cross routine baseline differences to provide a universal clinical basis for identifying populations at higher risk for inner ear ischemic injury.

The high consistency shown in the subgroup analyses provides extremely important systematic evidence for the etiology of SSNHL and strongly supplements previous lipid research in the field of deafness. In the past, many epidemiological studies on SSNHL and dyslipidemia were mostly limited to traditional indicators such as total cholesterol (TC), triglycerides (TG), or low-density lipoprotein (LDL-C), but the results often showed significant discrepancies (9, 13, 14). Therefore, this study introduced the ApoB/ApoA ratio, which is widely recognized as a more accurate reflection of the “pathogenic lipid balance” (10, 11). Through multivariable and matching models, its excellent value in predicting inner ear ischemic injury was confirmed. In previous clinical studies, whether age and sex could modify the effects of risk factors has always been a concern. Some studies suggest that the natural decline of vascular endothelial function with age may increase the susceptibility of inner ear microcirculation to ischemia (5). Meanwhile, some scholars have pointed out that differences in sex and estrogen levels may regulate the vascular endothelium, leading to potential differences in disease risk (15, 16). However, the subgroup analysis of this study (P for interaction for age and sex were 0.69 and 0.17, respectively) clearly showed that the pathogenic risk brought by a high ApoB/ApoA ratio has no significant statistical difference between young/middle-aged and middle-aged/elderly individuals, or between males and females. This is consistent with some clinical observations that the damage of metabolic risk factors to inner ear microcirculation is often independent of routine age and sex restrictions.

More importantly, the subgroup analysis of basic metabolic diseases revealed critical insights. In otolaryngology, hypertension and diabetes are widely recognized as classic risk factors for inner ear microvascular disease leading to SSNHL (14, 17). It is easy to suspect whether the hearing loss risk caused by lipid abnormalities is merely a “bystander effect” in patients with diabetes or hypertension. This study conducted strict stratification, showing that even in “apparently healthy” subjects without hypertension (OR = 12.16) and without diabetes (OR = 8.45), an elevated ApoB/ApoA ratio still led to a dramatic increase in the risk of SSNHL, with no significant interaction (P > 0.05). This systematic analysis not only aligns with otological clinical observations emphasizing that lipid toxicity is independent of blood glucose and blood pressure (17), but also strongly proves that dyslipidemia driven by an ApoB/ApoA imbalance is not merely an “accessory” to hypertension or diabetes. Instead, it is an independent factor associated with the ischemic process of the inner ear microcirculation, potentially preceding clinically diagnosed metabolic diseases (6).

We hypothesize that the potential pathological mechanisms by which a high ApoB/ApoA ratio contributes to SSNHL may be mainly related to the highly specialized microvascular anatomy of the inner ear and its presumed sensitivity to lipid toxicity. The blood supply to the cochlea relies completely on the labyrinthine artery, a small terminal artery, and the inner ear completely lacks a collateral circulation network (18, 19). ApoB represents all atherogenic lipoprotein particles, while ApoA is the core component of protective high-density lipoprotein (HDL) that maintains endothelial function and reverse cholesterol transport (10, 20). A significantly elevated ApoB/ApoA ratio indicates an overload of pathogenic lipid particles in the body (21). In the systemic vascular system, these excessive lipids easily invade and are retained in the subendothelium, triggering intense local oxidative stress and pro-inflammatory responses (22, 23). Based on the lack of collateral circulation in the labyrinthine artery, it is reasonable to speculate that this systemic lipid toxicity also affects the cochlea. Highly pathogenic lipid particles are hypothesized to undergo similar deposition and inflammatory cascades in the microvascular network of the stria vascularis, thereby potentially disrupting the extremely fragile microcirculatory homeostasis of the inner ear (18). It is biologically plausible that this lipid toxicity could contribute to endothelial dysfunction, a condition that has been repeatedly confirmed in multiple studies testing endothelial function in SSNHL patients (18, 24). Endothelial cell damage reduces nitric oxide (NO) synthesis and increases endothelin-1 (ET-1) release, which might induce abnormal spasms in the cochlear microvessels (25). A lack of protective ApoA may weaken the antioxidant and antithrombotic barriers of blood vessels (26, 27). In addition, lipid metabolic imbalances are often accompanied by a deterioration in hemorheological indicators, such as increased whole blood viscosity and decreased red blood cell deformability. Based on this, we have reason to speculate that this hypercoagulable state is highly likely to induce microthrombosis in the narrow capillaries of the stria vascularis, thereby triggering ischemic injury. Because cochlear hair cells have extremely poor tolerance to hypoxia, we postulate that the microvascular spasms and microthrombosis associated with lipid imbalance could lead to local energy metabolism failure, ultimately manifesting clinically as a sudden, cliff-like drop in hearing.

Although this study used a relatively large sample size and advanced statistical methods like PSM to enhance the robustness of the conclusions, some limitations remain. First, this is a retrospective cross-sectional design. While it confirms a strong association, it cannot establish an absolute causal relationship; prospective cohort studies are needed. Second, the data originated from a single clinical center, and the sample mainly represents the Chinese population in a specific region. The generalizability of the findings requires further confirmation through multi-center clinical studies. Third, this study mainly relied on a single biochemical test upon patient admission and failed to dynamically monitor apolipoprotein levels longitudinally before the onset and during the treatment. This limits an in-depth discussion on the relationship between lipid fluctuations and hearing recovery prognosis. Fourth, detailed audiometric data, such as specific frequency thresholds and audiometric configurations (e.g., ascending, descending, or flat curves), were not systematically extracted as structured quantitative data from the EMR system. Consequently, we were unable to perform sub-analyses stratifying the ApoB/ApoA ratio by specific hearing loss severity grades or audiometric curve types, limiting our ability to fully explore the clinical heterogeneity within the SSNHL group. Finally, although we fully adjusted for major baseline confounders, some relevant factors—such as smoking history, lifestyle habits, and crucially, the prior use of statins or other lipid-lowering medications—were not included in the models due to the retrospective nature of the EMR data. Because statins directly modify apolipoprotein profiles, this omission could introduce residual confounding. To quantify the potential impact of such unmeasured confounders, we calculated the E-value. The E-value for our fully adjusted model (OR = 9.34) was 18.16, with a lower confidence limit of 7.32. This indicates that an unmeasured confounder (e.g., statin use) would need to be associated with both a high ApoB/ApoA ratio and SSNHL risk by a risk ratio of at least 18.16 to completely explain away the observed association, and by 7.32 to shift the confidence interval to include the null. Given these substantially high thresholds, it is highly unlikely that unmeasured confounding alone could invalidate our primary findings, suggesting our results are robust. Future studies should attempt to collect and adjust for these variables to further refine the risk assessment system for inner ear diseases. Furthermore, caution is warranted when interpreting the magnitude of the odds ratios in certain stratified analyses (e.g., the diabetes and 55–85 years age subgroups). The extremely wide confidence intervals observed in these subgroups indicate statistical instability, which is likely attributable to data sparsity and reduced sample sizes upon stratification. While the overall interaction tests suggest a consistent positive trend across subgroups, the exact point estimates within these specific small subgroups possess limited interpretability.

## Conclusion

This study clearly demonstrates that an elevated ApoB/ApoA ratio is significantly associated with an increased risk of sudden sensorineural hearing loss (SSNHL). Rigorous stratified analyses confirm that the hearing loss risk caused by this lipid metabolism imbalance is independent of classic cardiovascular comorbidities such as hypertension and diabetes. The underlying pathological mechanisms may involve lipid toxicity caused by an overload of highly pathogenic lipid particles (ApoB) and a lack of protective lipoproteins (ApoA), which is hypothesized to contribute to endothelial dysfunction, abnormal spasms, and microthrombosis in the terminal microvascular network of the inner ear. This study highlights the value of combining clinical indicators with an epidemiological perspective. The ApoB/ApoA ratio has the potential to serve as a novel, independent clinical biomarker for the risk stratification and clinical assessment of SSNHL.

## Data Availability

The raw data supporting the conclusions of this article will be made available by the authors, without undue reservation.

## Supplementary material

Supplementary Table 1 Baselines characteristics of participants after PSM.

Supplementary Table 2 Baselines characteristics of participants after IPTW.
