# Supplementary material for: Elevated ApoB/ApoA ratio is associated with increased risk of sudden sensorineural hearing loss
Source: Front Endocrinol (Lausanne). 2026 Oct 15;17:1955992. doi: 10.3389/fendo.2026.1955992 (PMC13716602; doi:10.3389/fendo.2026.1955992)
Supplement: Supplementary file 1 [file Table1.docx]

Supplementary Table 1. Baselines characteristics of participants after PSM.

| **Characteristic** | **Normal** | **Hearing loss** | **P value** |
| --- | --- | --- | --- |
| **N** | **456** | **456** |  |
| Age(years) | 52.48 ± 12.92 | 56.96 ± 14.71 | <0.0001 |
| BUN(mmol/L) | 6.44 ± 3.76 | 6.16 ± 1.73 | 0.1536 |
| UA(umol/L) | 351.21 ± 98.85 | 317.63 ± 94.97 | <0.0001 |
| Cholesterol(mmol/L) | 5.07 ± 1.04 | (5.08 ± 1.00 | 0.9739 |
| TBIL(μmol/L) | 12.57 ± 5.62 | 14.40 ± 6.13 | <0.0001 |
| TG(mmol/L) | 1.60 ± 0.77 | 1.29 ± 0.90 | <0.0001 |
| Cre(μmol/L) | 98.28 ± 78.73 | 59.02 ± 15.13 | <0.0001 |
| FPG(mmol/L) | 5.98 ± 1.72 | 6.25 ± 2.07 | 0.0285 |
| ALT (U/L) | (24.77 ± 17.23 | 22.46 ± 20.49 | 0.0653 |
| AST (U/L) | 21.25 ± 9.35 | 23.00 ± 12.54 | 0.0172 |
| BMI(kg/m^2^) | 24.99 ± 3.72 | 24.15 ± 3.43 | 0.0004 |
| ApoB/ApoA | 0.71 ± 0.20 | 0.70 ± 0.25 | 0.783 |
| Gender(%) |  |  | 0.0022 |
| Male | 304 (66.7) | 258 (56.6) |  |
| Female | 152 (33.3) | 198 (43.4) |  |
| High Blood Pressure(%) |  |  | <0.0001 |
| No | 260 (57) | 365 (80) |  |
| Yes | 196 (43) | 91 (20) |  |
| Diabetes(%) |  |  | 0.8726 |
| No | 358 (78.5) | 355 (77.9) |  |
| Yes | 98 (21.5) | 101 (22.1) |  |

Supplementary Table 2. Baselines characteristics of participants after IPTW.

|  | **Low(0.**13-0.58) | **Middle(0.**58-0.78) | **High(0.**78-1.34) | P value |
| --- | --- | --- | --- | --- |
| Age(years) | 49.78 ± 10.79 | 54.54 ± 13.52 | 54.34 ± 14.28 | <0.0001 |
| BUN(mmol/L) | 6.00 ± 1.78 | 6.22 ± 2.79 | 6.35 ± 2.46 | 0.0691 |
| UA(umol/L) | 404.90 ± 100.53 | 330.17 ± 92.40 | 331.11 ± 103.65 | <0.0001 |
| Cholesterol(mmol/L) | 7.82 ± 3.16 | 5.08 ± 0.92 | 5.10 ± 0.93 | <0.0001 |
| TBIL(μmol/L) | 9.72 ± 5.21 | 13.10 ± 6.74 | 13.11 ± 5.53 | <0.0001 |
| TG(mmol/L) | 1.65 ± 1.38 | 1.45 ± 0.82 | 1.46 ± 0.76 | 0.0084 |
| Cre(μmol/L) | 74.69 ± 46.59 | 76.30 ± 35.16 | 81.05 ± 38.92 | 0.1021 |
| FPG(mmol/L) | 5.90 ± 1.78 | 6.08 ± 1.80 | 6.06 ± 1.92 | 0.2748 |
| ALT (U/L) | 26.26 ± 16.94 | 22.34 ± 13.95 | 24.87 ± 22.77 | 0.0085 |
| AST (U/L) | 23.18 ± 7.78 | 21.50 ± 7.82 | 23.01 ± 14.78 | 0.0498 |
| BMI(kg/m^2^) | 26.64 ± 3.30 | 24.43 ± 3.62 | 24.33 ± 3.56 | <0.0001 |
| Gender(%) |  |  |  | <0.0001 |
| Male | 77.17 | 57.48 | 58.33 |  |
| Female | 22.83 | 42.52 | 41.67 |  |
| High Blood Pressure(%) |  |  |  | <0.0001 |
| No | 83.84 | 67.81 | 68.81 |  |
| Yes | 16.16 | 32.19 | 31.19 |  |
| Diabetes(%) |  |  |  | 0.0302 |
| No | 85.58 | 79.2 | 80.66 |  |
| Yes | 14.42 | 20.8 | 19.34 |  |
| Hearing loss(%) |  |  |  | <0.0001 |
| No | 69.37 | 58.75 | 51.6 |  |
| Yes | 30.63 | 41.25 | 48.4 |  |
